# Effect of Failed Debridement, Antibiotics, and Implant Retention on Subsequent Two-Stage Reimplantation for Periprosthetic Joint Infection Following Total Knee Arthroplasty: A Retrospective Cohort Study

**DOI:** 10.3390/jcm15124563

**Published:** 2026-06-12

**Authors:** Dong Hwi Kim, GwangChul Lee, Ba Woo Ko, Jae Hwan Lim, Suenghwan Jo

**Affiliations:** 1 College of Medicine, Chosun University, Gwangju 61452, Republic of Korea; oskdh@chosun.ac.kr (D.H.K.); leekci@chosun.ac.kr (G.L.); 2 Department of Orthopedic Surgery, Chosun University Hospital, Gwangju 61453, Republic of Korea; popo-04@hanmail.net; 3 Department of Orthopaedic Surgery, Daejung Hospital, Gwangju 61473, Republic of Korea; eternul@naver.com

**Keywords:** periprosthetic joint infection, total knee arthroplasty, debridement, antibiotics, and implant retention, two-stage revision, treatment failure

## Abstract

**Background**: Periprosthetic joint infection (PJI) following total knee arthroplasty (TKA) is a serious complication associated with substantial morbidity and healthcare costs. Although two-stage revision arthroplasty is widely accepted as a standard treatment, debridement, antibiotics, and implant retention (DAIR) is frequently attempted as a less invasive initial strategy. However, the impact of failed DAIR on the outcomes of subsequent two-stage revision remains controversial. **Methods**: This retrospective cohort study included patients who underwent two-stage revision arthroplasty for PJI following TKA at a single institution between 2005 and 2019, involving 84 knees at an average follow-up of 5.1 ± 3.3 years. Outcomes were compared between patients with a history of failed DAIR (F-DAIR group, *n* = 23) and those who underwent direct two-stage revision without prior procedures (DTSR group, *n* = 61). Treatment failure rates and associated risk factors were analyzed. **Results**: There was no significant difference in treatment failure rates between the F-DAIR and DTSR groups (4.3% vs. 9.8%, respectively; *p* = 0.668). Cox regression suggested that PJI type (acute vs. chronic) and diabetes mellitus were associated with treatment failure. **Conclusions**: In this retrospective cohort, prior failed DAIR was not associated with a statistically significant reduction in the success of subsequent two-stage revision arthroplasty. These findings may support consideration of DAIR as an initial treatment option in carefully selected patients; however, cautious interpretation is warranted because of the retrospective design, limited sample size, baseline imbalance, and small number of failure events. Therefore, the findings should be considered hypothesis-generating rather than definitive comparative evidence.

## 1. Introduction

Total knee arthroplasty (TKA) is one of the most successful orthopedic procedures for end-stage knee osteoarthritis, providing significant pain relief and functional improvement [1]. However, periprosthetic joint infection (PJI) remains one of the most devastating complications following TKA, occurring in 1–2% of primary procedures [2]. PJI is associated with significant morbidity, mortality, healthcare costs, and compromised functional outcomes, making it a major concern for both patients and surgeons [3,4]. Host-related factors, including diabetes mellitus, may further impair infection control and increase the risk of treatment failure after surgical treatment for PJI.

Two-stage revision arthroplasty, involving prosthesis removal, antibiotic spacer placement, and subsequent reimplantation, is widely accepted as a standard treatment for chronic PJI, with reported success rates ranging from 80% to 100% [5]. However, this approach has significant limitations, including multiple surgical procedures, substantial perioperative morbidity, prolonged treatment duration, loss of bone stock, and decreased mobility between stages [6,7,8].

Debridement, antibiotics, and implant retention (DAIR) has emerged as a less invasive alternative for selected cases of PJI, particularly for acute postoperative and acute hematogenous infections [9,10,11]. DAIR offers several advantages, including reduced perioperative morbidity, faster recovery, preservation of bone stock, and lower healthcare costs. However, success rates for DAIR vary widely, ranging from 50% to 80%, depending on patient selection, timing of intervention, and infection characteristics [6,9,10,11,12,13,14].

When DAIR fails to eradicate infection, patients typically require two-stage revision arthroplasty as a salvage procedure [15]. A critical clinical question arises: does the failure of DAIR compromise the success of subsequent two-stage revision? This question has important implications for treatment algorithms and patient counseling. Some studies have suggested that prior failed DAIR may negatively impact subsequent revision outcomes due to increased bacterial biofilm formation, tissue damage, and antibiotic resistance [16,17,18,19]. Conversely, other studies have found no significant difference in outcomes between patients with and without prior DAIR [6,16,20,21,22].

The existing literature presents conflicting results, with variations in study design, patient populations, DAIR protocols, and outcome definitions contributing to this uncertainty. Furthermore, most studies have been conducted in Western populations, with limited data from Asian cohorts. Understanding the impact of failed DAIR on subsequent two-stage revision outcomes is essential for developing evidence-based treatment algorithms and optimizing patient outcomes. Differences in healthcare systems, referral patterns, microbiological epidemiology, antibiotic usage, and surgical strategies may influence the generalizability of existing findings to Asian populations. Therefore, additional data from Asian cohorts are needed to contextualize treatment outcomes. Standardized diagnostic criteria and outcome definitions are essential for studies evaluating PJI treatment outcomes. The Musculoskeletal Infection Society (MSIS) criteria provide a widely accepted diagnostic framework incorporating clinical findings, inflammatory markers, microbiological cultures, and intraoperative findings; the MSIS outcome reporting tool further classifies results according to infection control, need for reoperation, antibiotic suppression, spacer retention, arthrodesis, amputation, and mortality. Use of such standardized criteria is particularly important because reported success rates after DAIR or two-stage revision vary substantially depending on how treatment failure is defined [23,24,25,26].

The purpose of this retrospective cohort study was to compare the success rates of two-stage revision arthroplasty for PJI following primary TKA between patients with prior failed DAIR (F-DAIR group) and those without prior procedures (DTSR group) in a Korean single-institution cohort, and to identify predictive factors associated with treatment failure in this population.

## 2. Materials and Methods

### 2.1. Data Collection

This retrospective cohort study was conducted at Chosun University Hospital, a tertiary referral center in Gwangju, Republic of Korea, and specifically evaluated a Korean patient cohort treated for PJI following primary TKA. We reviewed medical records of all patients who underwent two-stage revision arthroplasty for PJI following primary TKA between February 2005 and December 2019. All patients met the Musculoskeletal Infection Society (MSIS) diagnostic criteria for PJI, which incorporate clinical findings, serum inflammatory markers, microbiological cultures, and intraoperative findings [5]. Telephone follow-up was conducted in December 2020 to assess outcomes and ensure a minimum one-year follow-up for all patients, except for those who died within one year. The study was approved by the Institutional Review Board of Chosun University Hospital (IRB No. 2021-08-018).

This study was reported in accordance with the Strengthening the Reporting of Observational Studies in Epidemiology (STROBE) recommendations where applicable [27].

**Figure 1:**
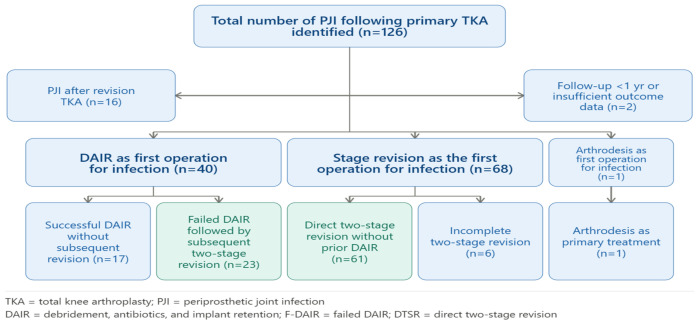
Flowchart outlining patient inclusion and reasons for exclusions. The revised flowchart provides detailed sequential exclusion criteria to improve transparency of patient selection.

A total of 126 patients with PJI following primary TKA were initially identified. After applying inclusion and exclusion criteria, 84 patients were included in the final analysis (Figure 1). Patients were divided into two groups: the F-DAIR group (*n* = 23) consisted of patients who underwent failed DAIR followed by two-stage revision, and the DTSR (Direct Two-Stage Revision) group (*n* = 61) consisted of patients who underwent two-stage revision as the primary treatment for PJI without prior DAIR.

Patients were eligible for inclusion if they met all of the following criteria: (1) diagnosis of periprosthetic joint infection following primary total knee arthroplasty according to the Musculoskeletal Infection Society (MSIS) criteria; (2) treatment with planned two-stage revision arthroplasty between February 2005 and December 2019; (3) completion of both first-stage explantation with antibiotic-loaded cement spacer insertion and second-stage reimplantation; (4) availability of sufficient clinical, microbiological, operative, and follow-up data for outcome assessment; and (5) minimum follow-up duration of one year after treatment initiation, except for patients who died within one year.

Patients were excluded if they had: (1) PJI after revision TKA rather than primary TKA, to reduce heterogeneity associated with prior revision procedures; (2) successful DAIR without subsequent two-stage revision, because the study objective was to evaluate outcomes after failed DAIR requiring salvage revision; (3) incomplete two-stage revision, including permanent spacer retention or failure to undergo reimplantation; (4) arthrodesis, resection arthroplasty, or amputation as the definitive initial treatment strategy; (5) follow-up shorter than one year without documented treatment failure or death; or (6) insufficient medical records to determine infection status, treatment pathway, or treatment outcome.

Patient demographics, comorbidities, infection characteristics, microbiological data, surgical details, and outcomes were extracted from medical records. Demographic variables included age, sex, body mass index (BMI), American Society of Anesthesiologists (ASA) grade, and comorbidities (diabetes mellitus, rheumatoid arthritis, chronic obstructive pulmonary disease, chronic kidney disease, liver cirrhosis, and cardiovascular disease). Infection characteristics included PJI type (acute postoperative, acute hematogenous, or chronic), duration of symptoms, implant age at time of infection, and microbiological culture results.

### 2.2. Surgical Methods

#### 2.2.1. DAIR Procedure (F-DAIR Group)

In the F-DAIR group, 23 patients underwent a total of 27 DAIR procedures before two-stage revision. Nineteen patients (82.6%) underwent DAIR once, while four patients (17.4%) underwent DAIR twice. Fifteen DAIR procedures (55.6%) were performed at our institution, while 12 procedures (44.4%) were performed at outside institutions before referral. DAIR procedures included arthroscopic synovectomy (15 procedures, 55.6%), open synovectomy (1 procedure, 3.7%), and open synovectomy with liner exchange (11 procedures, 40.7%).

DAIR procedures typically involve thorough debridement of infected tissue, synovectomy, copious irrigation (minimum 10 L of antibiotic-mixed solution), and polyethylene liner exchange in an open DAIR procedure. Multiple arthroscopic portals (supralateral, anteromedial, anterolateral, posteromedial, and posterolateral) were used for comprehensive joint access, especially including the posterior compartment during arthroscopic procedures.

The mean interval from DAIR to first-stage revision was 3.91 months (±4.32, range: 0–17 months). Twenty-two patients (95.6%) underwent first-stage revision within one year of DAIR, while one patient (4.3%) had a 17-month interval. This interval was not predefined but reflected real-world referral patterns, variability in initial treatment response, and differences in the timing at which persistent or recurrent infection was clinically recognized.

#### 2.2.2. Two-Stage Revision Arthroplasty

All patients in both groups underwent standardized two-stage revision arthroplasty. The first stage involved complete removal of all prosthetic components, thorough debridement of infected and necrotic tissue, multiple tissue and fluid samples for culture (minimum 5 samples), copious irrigation, and placement of an antibiotic-loaded cement spacer. The spacer was fabricated using 40 g of bone cement (Palacos, Heraeus Medical GmbH, Wehrheim, Germany) mixed with 2 g of vancomycin per 40 g of cement.

Following first-stage surgery, patients received intravenous antibiotics for 6–10 weeks based on culture results and antibiotic sensitivity without an antibiotic-free period. If the culture test showed negative results, broad-spectrum antibiotics, typically vancomycin or teicoplanin, were continued until the second stage of the arthroplasty. All patients were advised to begin quadriceps strengthening exercises immediately after surgery. Additionally, patients who received a movable cement spacer were permitted to perform a range of motion exercises and weight-bearing ambulation, as tolerated. Infection control was assessed by clinical examination, inflammatory markers (erythrocyte sedimentation rate [ESR] and C-reactive protein [CRP]), and joint aspiration when indicated. Second-stage reimplantation was performed when clinical signs of infection resolved and inflammatory markers normalized or showed significant improvement.

The mean interval between first and second stages was 62.80 days (±34.39). Second-stage surgery involved spacer removal, repeat debridement, multiple tissue cultures, and implantation of a new prosthesis with antibiotic-loaded cement fixation. Patients received only prophylactic antibiotics during the operation and for one day postoperatively, except in cases of fungal infection, for which oral antifungal agents were prescribed for six months.

### 2.3. Outcome Measurement

The primary outcome was treatment failure, defined according to the MSIS outcome reporting tool (Table 1) [5]. Treatment success was defined as infection control without continued antibiotic suppression (Tier 1) or with antibiotic suppression (Tier 2). Treatment failure was defined as the need for reoperation for infection (Tier 3D), death within one year of PJI treatment initiation (Tier 4A), or persistent infection requiring additional surgical intervention [28].

Secondary outcomes included: (1) overall survival free from treatment failure, (2) mortality rates (PJI-related and all-cause), (3) microbiological profiles, and (4) predictive factors for treatment failure.

### 2.4. Statistical Analysis

Continuous variables were expressed as means with standard deviations or ranges and compared between groups using Student’s *t*-test. Categorical variables were expressed as frequencies with percentages and compared using chi-square test or Fisher’s exact test as appropriate. Kaplan–Meier survival curves were constructed to compare failure-free survival between groups, with log-rank test used for comparison. Cox proportional hazards regression analysis was performed to identify independent predictors of treatment failure, with results expressed as hazard ratios (HR) with 95% confidence intervals (CI). Variables with *p* < 0.10 in univariate analysis were included in multivariate models. All statistical analyses were performed using SPSS version 20.0 (IBM Corp., Armonk, NY, USA). A two-tailed *p*-value < 0.05 was considered statistically significant. Because substantial baseline differences existed between groups, particularly regarding acute versus chronic infection status, all comparative findings were interpreted cautiously. Cox proportional hazards regression was used as an exploratory method to partially account for potential confounding factors; however, comprehensive adjustment for all potential confounders was not feasible because of the limited number of failure events.

Because treatment allocation was not randomized and substantial baseline imbalance existed, particularly in infection chronicity, formal causal inference was not attempted. Propensity-score matching, inverse-probability weighting, and stratified subgroup analyses were considered but were not performed because the F-DAIR group and the number of failure events were too small to support stable estimates [29]. Therefore, the Cox regression analysis was used only to explore potential associations rather than to establish independent causal effects.

## 3. Results

### 3.1. Patient Demographics

The demographic characteristics of the two groups are summarized in Table 2. The mean age was 69.78 years (range: 38–84) in the F-DAIR group and 71.64 years (range: 48–83) in the DTSR group (*p* = 0.409). There were no significant differences between groups in terms of sex distribution (*p* = 0.271), ASA grade (*p* = 0.300), BMI (*p* = 0.228), or most comorbidities.

However, significant differences were observed in infection type distribution (*p* = 0.001). The F-DAIR group had a higher proportion of acute infections (acute postoperative: 43.5%, acute hematogenous: 39.1%) compared to the DTSR group (acute postoperative: 11.5%, acute hematogenous: 36.1%), while the DTSR group had a higher proportion of chronic infections (52.5% vs. 17.4%).

The mean implant age at time of infection was 26.65 months (range: 1–173) in the F-DAIR group and 45.07 months (range: 2–203) in the DTSR group, showing a trend toward longer implant duration in the DTSR group (*p* = 0.097). The mean interval between first and second stages was 65.23 days (range: 27–279) in the F-DAIR group and 56.34 days (range: 40–101) in the DTSR group (*p* = 0.149).

### 3.2. Microbiology

Microbiological profiles differed significantly between groups (Table 3). Culture-negative rates were significantly higher in the DTSR group (54.1%, 33/61) compared to the F-DAIR group (17.4%, 4/23) (*p* = 0.003). Conversely, antibiotic-resistant bacteria were significantly more common in the F-DAIR group (47.8%, 11/23) compared to the DTSR group (13.1%, 8/61) (*p* = 0.001).

The most common organism overall was methicillin-resistant *Staphylococcus epidermidis* (MRSE), accounting for 15.5% (13/84) of cases. MRSE was significantly more prevalent in the F-DAIR group (39.1%, 9/23) compared to the DTSR group (6.6%, 4/61) (*p* = 0.001). Other common organisms included methicillin-resistant *Staphylococcus aureus* (MRSA) (7.1%, 6/84), *Escherichia coli* (7.1%, 6/84), and methicillin-sensitive *Staphylococcus aureus* (MSSA) (6.0%, 5/84).

### 3.3. Treatment Outcomes

Treatment outcomes according to the MSIS classification are presented in Table 4. The overall treatment success rate (Tier 1) was 78.6% (66/84). The failure rate was 4.3% (1/23) in the F-DAIR group and 9.8% (6/61) in the DTSR group, with no statistically significant difference between groups (*p* = 0.668).

In the F-DAIR group, 20 patients (87.0%) achieved Tier 1 outcome (infection control without antibiotic suppression), 1 patient (4.3%) experienced treatment failure requiring reoperation (Tier 3D), and 2 patients (8.7%) died more than one year after treatment initiation from causes unrelated to PJI (Tier 4B).

In the DTSR group, 46 patients (75.4%) achieved Tier 1 outcome, 2 patients (3.3%) required aseptic revision more than one year after treatment (Tier 3B), 2 patients (3.3%) experienced treatment failure requiring reoperation (Tier 3D), 2 patients (3.3%) died within one year of treatment initiation (Tier 4A), and 9 patients (14.8%) died more than one year after treatment from causes unrelated to PJI (Tier 4B).

### 3.4. Survival Analysis

**Figure 2:**
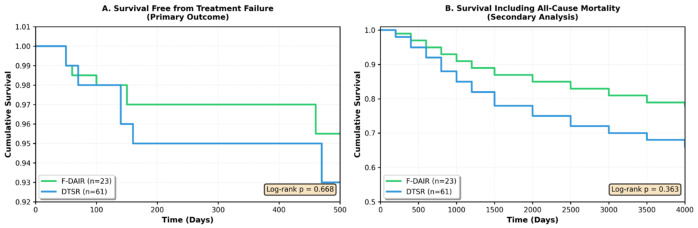
Kaplan–Meier survival curves comparing the F-DAIR and DTSR groups.

Kaplan–Meier survival analysis showed no statistically significant difference in failure-free survival between the F-DAIR and DTSR groups (log-rank *p* = 0.668). The estimated cumulative survival free from treatment failure at 5 years was 95.7% in the F-DAIR group and 90.2% in the DTSR group (Figure 2).

In the primary survival analysis, the F-DAIR group achieved cumulative failure-free survival of 95.7% at 5 years, with a single failure event at approximately 15 months post-reimplantation. The DTSR group achieved 90.2% failure-free survival at 5 years, with six failure events distributed across the follow-up period. The log-rank test showed no statistically significant difference between the two curves (*p* = 0.668). This finding should not be interpreted as proof of equivalence because of the small sample size and limited number of events.

In a secondary analysis incorporating all-cause mortality, the F-DAIR group achieved 91.3% survival at 5 years (3 total events: 1 infection-related failure, 2 non-PJI deaths), and the DTSR group achieved 75.4% (15 events: 4 infection-related failures, 2 PJI-related deaths, 9 deaths from other causes). No significant difference was observed between groups in this analysis either (*p* = 0.363). Both groups maintained high early survival (>95% at 1 year), and failure events occurred predominantly within the first 2 years, consistent with typical recurrent PJI timelines. The curves showed no progressive divergence over time, suggesting no clear delayed disadvantage associated with prior DAIR in this cohort, although the small number of events limits this interpretation.

Five-year mortality rates were also similar between groups. PJI-related mortality at 5 years was 0% in the F-DAIR group and 3.3% in the DTSR group (*p* = 1.000). All-cause mortality at 5 years was 8.7% in the F-DAIR group and 13.1% in the DTSR group (*p* = 0.720). The slightly higher all-cause mortality in the DTSR group likely reflects the older age and higher proportion of chronic infections with associated comorbidities in this group, rather than any treatment-related effect.

### 3.5. Predictive Factors

Exploratory Cox proportional hazards regression analysis suggested potential associations with treatment failure (Table 5). In this analysis, PJI type (acute vs. chronic) was associated with treatment failure, with chronic infections showing higher failure risk (HR = 0.028, 95% CI: 0.002–0.527, *p* = 0.017). Diabetes mellitus also showed an association with treatment failure (HR = 12.809, 95% CI: 1.217–134.769, *p* = 0.034). These findings should be interpreted cautiously because only seven failure events were observed.

Prior DAIR showed a trend toward lower failure rates but did not reach statistical significance (HR = 0.043, 95% CI: 0.002–1.039, *p* = 0.053). Other factors including age, culture results, antibiotic resistance, rheumatoid arthritis, liver cirrhosis, implant age, and ASA score were not significant predictors of treatment failure.

Given the limited number of failure events, the Cox regression analysis should be interpreted as exploratory and hypothesis-generating. The wide confidence intervals observed for several covariates likely reflect event scarcity and model instability, and the hazard ratio estimates should therefore be interpreted cautiously.

Because only seven treatment-failure events were observed, the estimates in this table should be interpreted as exploratory associations rather than robust adjusted effect estimates.

## 4. Discussion

This retrospective cohort study provides clinical data regarding the impact of failed DAIR on subsequent two-stage revision outcomes for PJI following TKA, involving 84 knees at an average follow-up of 5.1 years. Our principal finding is that prior failed DAIR was not associated with significantly worse outcomes after subsequent two-stage revision arthroplasty in this cohort. The failure rate was 4.3% in the F-DAIR group compared to 9.8% in the DTSR group (*p* = 0.668), with no statistically significant difference in failure-free survival between groups. However, these findings should be interpreted cautiously because of the retrospective design, baseline imbalance, limited sample size, and small number of failure events. These findings are consistent with a recent systematic review and meta-analysis suggesting that prior failed DAIR does not uniformly compromise subsequent two-stage revision success, although a nonsignificant trend toward lower success and substantial between-study heterogeneity were noted. Notably, sensitivity analyses in that meta-analysis suggested that the estimated association can shift depending on study inclusion and the applied definition of success, underscoring the need for cautious interpretation [30]. Accordingly, the present findings should be interpreted primarily as hypothesis-generating observational data rather than definitive evidence that failed DAIR has no adverse effect on subsequent two-stage revision outcomes.

These findings may have potential clinical implications, as they support consideration of DAIR as an initial treatment strategy for appropriately selected patients while preserving subsequent two-stage revision as a salvage option if DAIR fails. The comparable outcomes between groups may be explained by several factors. First, the thorough debridement and complete prosthesis removal during first-stage revision effectively addresses any residual infection or biofilm formation from the failed DAIR procedure. Second, the prolonged antibiotic therapy (6–10 weeks [31]) between stages provides adequate time for infection eradication. Third, the standardized surgical protocol and careful patient selection in our institution may have contributed to favorable outcomes in both groups.

To further contextualize these findings, the exploratory Cox regression analysis suggested that PJI type and diabetes mellitus may be associated with treatment failure, but these variables should not be interpreted as definitive independent predictors because of the limited number of events. PJI type showed the strongest apparent association, with acute infections (both postoperative and hematogenous) having better outcomes compared with chronic infections (HR = 0.028, *p* = 0.017). This finding aligns with established principles of PJI management, as chronic infections are associated with mature biofilm formation, more extensive tissue damage, and often more virulent or resistant organisms [5,6].

In addition, diabetes mellitus was identified as another significant predictor of treatment failure (HR = 12.809, *p* = 0.034). This finding is consistent with extensive literature demonstrating that diabetes impairs wound healing, immune function, and infection control [2]. Diabetic patients have compromised neutrophil function, reduced tissue perfusion, and altered inflammatory responses, all of which contribute to increased infection risk and treatment failure. This finding emphasizes the importance of optimizing glycemic control before and after revision surgery, and suggests that diabetic patients may require more intensive monitoring and potentially longer antibiotic therapy. Preoperative glycemic assessment, including hemoglobin A1c (HbA1c) and perioperative blood glucose monitoring, may help identify patients at elevated risk of wound complications or recurrent infection. In diabetic patients undergoing staged revision, optimization of glycemic control before reimplantation, nutritional support, and multidisciplinary involvement of internal medicine or endocrinology specialists may be beneficial. Detailed glycemic parameters were not systematically collected in this study; future studies should investigate their prognostic value [32,33].

Interestingly, although the F-DAIR group had significantly higher rates of antibiotic-resistant organisms (47.8% vs. 13.1%, *p* = 0.001), particularly MRSE (39.1% vs. 6.6%, *p* = 0.001), this did not translate into higher failure rates. This paradoxical finding may be explained by several factors. First, the identification of resistant organisms during DAIR allowed for targeted antibiotic selection during subsequent two-stage revision [7]. Second, the mechanical removal of infected tissue and biofilm during first-stage revision may be more important than antibiotic susceptibility patterns [3]. Third, the use of high-dose local antibiotics in cement spacers achieves concentrations far exceeding minimum inhibitory concentrations, which may improve local antimicrobial activity even when in vitro susceptibility is reduced [8,34].

Similarly, the significantly higher culture-negative rate in the DTSR group (54.1% vs. 17.4%, *p* = 0.003) is noteworthy. This difference likely reflects the fact that many patients in the DTSR group received empiric antibiotics before culture sampling, either at outside institutions or during initial management. In contrast, patients in the F-DAIR group had organisms identified during the DAIR procedure, providing microbiological guidance for subsequent treatment. Despite this difference, culture-negative status was not a significant predictor of treatment failure in our Cox regression analysis, suggesting that empiric broad-spectrum antibiotic therapy can be effective when cultures are negative [7].

Taken together, our findings contribute to a growing but conflicting body of literature examining the impact of failed DAIR on subsequent revision outcomes. The existing studies show considerable heterogeneity in results, study design, patient populations, and outcome definitions, making direct comparisons challenging. Notably, contemporary evidence syntheses emphasize that reported success rates are highly sensitive to outcome definitions and study inclusion criteria, which may partly explain conflicting results across the literature [30]. This variability is further amplified by the retrospective nature of most studies and potential selection bias, as antecedent-DAIR cohorts inherently exclude DAIR successes and may over-represent more complex infections [13,30,35].

Sherrell et al. [6] reported one of the earliest studies on this topic, finding a 34% failure rate in patients undergoing two-stage revision after failed DAIR. However, their study included all DAIR procedures, regardless of where they were performed, and used a broad definition of failure that included any reoperation. Gardner et al. [36] reported a 42% failure rate in a small cohort of 19 patients with prior failed DAIR, suggesting that prior DAIR significantly compromised subsequent revision outcomes.

More recent studies have reported more favorable outcomes. Brimmo et al. [16] analyzed 750 patients and found 4-year failure rates of 8.7% in the F-DAIR group and 17.5% in the DTSR group, with no significant difference between groups. Nodzo et al. [17] reported similar failure rates of 17.8% in the F-DAIR group and 17.5% in the DTSR group (*p* = 0.95) in a cohort of 177 patients. Kim et al. [20] found failure rates of 20.0% in the F-DAIR group and 15.9% in the DTSR group (*p* = 0.22) in 138 patients, concluding that failed DAIR does not compromise subsequent staged revision success.

In contrast, Kavolus et al. reported in prosthetic hip infection that antecedent DAIR was associated with higher failure after subsequent two-stage reimplantation and greater treatment burden (including more operative episodes and longer hospitalization), underscoring that the impact of failed DAIR may vary by joint, host factors, and management pathways. Their findings also emphasize the importance of close surveillance after DAIR and timely transition to component removal when treatment response is inadequate, to avoid prolonged infection and escalating treatment burden [35].

Our study reports lower failure rates than most previous studies (4.3% in F-DAIR group, 9.8% in DTSR group), which may be attributed to several factors. First, we used a strict definition of failure based on the MSIS outcome reporting tool, focusing on infection-related failures within one year of treatment initiation. Many previous studies used broader failure definitions that included aseptic revisions, mechanical complications, or any reoperation, which may inflate failure rates. Second, our standardized surgical protocol, consistent antibiotic regimen, and careful patient selection may have contributed to favorable outcomes. Third, our relatively short mean follow-up period may not have captured late failures, though most infection-related failures occur within the first two years. In addition, we did not evaluate resource-utilization outcomes, which have been highlighted as potentially important even when infection-control endpoints are similar [28,35].

Conversely, some studies have reported negative impacts of prior DAIR. Rajgopal et al. [18] found a 2-year failure rate of 21.5% in the F-DAIR group compared to 14.6% in the DTSR group, though this difference was not statistically significant. Lizaur-Utrilla et al. [19] reported that prior debridement had a negative impact on functional outcomes of subsequent two-stage revision for early knee PJI, though they focused on functional outcomes rather than infection control.

Several factors may explain the heterogeneity in reported outcomes across studies. First, the definition and timing of DAIR vary considerably. Some studies include only arthroscopic debridement, while others include open procedures with or without liner exchange. The timing of DAIR relative to symptom onset also varies, with some studies including only acute infections while others include chronic infections. Second, the criteria for proceeding to two-stage revision after failed DAIR differ across institutions. Some centers proceed to revision quickly after DAIR failure, while others attempt multiple DAIR procedures. Third, surgical techniques, antibiotic protocols, and patient populations vary across institutions and geographic regions. Moreover, definitions of treatment success/failure differ substantially between studies, and even small changes in outcome criteria can materially alter reported success rates, limiting cross-study comparability [30].

Our study’s relatively favorable outcomes may also reflect patient selection bias. Patients in our F-DAIR group had predominantly acute infections (82.6% acute postoperative or hematogenous), which are known to have better outcomes with DAIR and subsequent revisions. In contrast, the DTSR group had a higher proportion of chronic infections (52.5%), which may have been deemed unsuitable for DAIR from the outset due to factors such as implant loosening, sinus tract formation, or prolonged symptom duration. This imbalance indicates confounding by indication, because patients selected for DAIR and those treated with direct two-stage revision differed in clinical presentation and likely in baseline prognosis. Therefore, direct comparison between the two treatment pathways should be interpreted cautiously.

Our findings have several important clinical implications for the management of PJI following TKA. First and most importantly, the present findings may provide supportive evidence that attempting DAIR as an initial treatment strategy in appropriately selected patients is not necessarily associated with worse outcomes after subsequent two-stage revision if DAIR fails. This finding supports a stepwise treatment algorithm that begins with less invasive options (DAIR) for acute infections before proceeding to more extensive procedures (two-stage revision) when necessary. Nevertheless, because some series suggest that failed DAIR may increase treatment burden and compromise subsequent outcomes in certain settings [35], close surveillance after DAIR is warranted, and timely conversion to component removal should be considered when clinical, serologic, or radiographic indicators fail to improve.

The decision to attempt DAIR should be based on established criteria, including: (1) acute infection (symptom duration < 3 weeks from symptom onset), (2) well-fixed implants without evidence of loosening, (3) intact soft tissue envelope without sinus tracts, (4) organism susceptible to oral biofilm-active antibiotics, and (5) healthy host without significant immunocompromise [9,10,11]. Our finding that acute infections had significantly better outcomes than chronic infections (HR = 0.028, *p* = 0.017) reinforces the importance of infection timing in treatment selection [3,7].

Third, the high rate of antibiotic-resistant organisms in the F-DAIR group (47.8%, particularly MRSE at 39.1%) highlights the importance of obtaining adequate cultures during DAIR procedures and using this microbiological information to guide subsequent treatment. When DAIR fails and two-stage revision is planned, surgeons should review prior culture results and antibiotic sensitivities to optimize antibiotic selection for cement spacers and systemic therapy. The use of vancomycin in cement spacers, as employed in our protocol, provides broad coverage against resistant Gram-positive organisms including MRSE and MRSA [7,37].

Fourth, the high culture-negative rate in the DTSR group (54.1%) emphasizes the importance of obtaining cultures before initiating antibiotic therapy whenever possible. When patients present with suspected PJI, antibiotics should be withheld until adequate tissue and fluid samples are obtained for culture, unless the patient is septic or hemodynamically unstable. For culture-negative cases, empiric broad-spectrum antibiotic therapy targeting common PJI organisms (including resistant staphylococci) is appropriate, with consideration of extended culture techniques or molecular diagnostic methods when available.

Fifth, our findings support the use of standardized protocols for two-stage revision, including thorough debridement, adequate antibiotic therapy duration (6–10 weeks [31] between stages), and careful assessment of infection control before reimplantation. The consistency of our surgical technique and antibiotic protocol likely contributed to favorable outcomes in both groups.

Finally, patient selection and shared decision-making are critical. Patients should be counseled about the potential need for multiple procedures, the possibility of DAIR failure, and the overall treatment timeline. For patients with acute infections and favorable characteristics (well-fixed implants, healthy host, susceptible organisms), DAIR may be considered as a potential initial approach with the understanding that two-stage revision remains an effective salvage option if DAIR fails. For patients with chronic infections, implant loosening, immunocompromise, or resistant organisms, proceeding directly to two-stage revision may be more appropriate.

This study has several strengths. First, it represents one of the largest single-institution cohorts comparing outcomes of two-stage revision with and without prior failed DAIR in a Korean patient population, providing data from an Asian cohort underrepresented in the existing literature. Second, we used standardized surgical techniques and antibiotic protocols throughout the study period, reducing variability in treatment approaches. Third, we employed the MSIS outcome reporting tool, which provides a standardized, internationally recognized framework for outcome assessment. Fourth, we performed Cox regression analysis to explore potential predictors of treatment failure, providing insights beyond simple outcome comparisons. Fifth, we achieved complete follow-up through telephone interviews in December 2020, minimizing loss to follow-up.

However, several limitations must be acknowledged. First, the retrospective design introduces inherent biases and limitations in data quality. Most importantly, selection bias and confounding by indication are major concerns, as the decision to attempt DAIR versus proceed directly to two-stage revision was based on surgeon judgment and clinical presentation rather than randomization. The F-DAIR group included a larger proportion of acute infections with generally favorable characteristics for implant retention, whereas the DTSR group included more chronic infections, which likely reflects real-world treatment selection rather than comparable baseline risk. Because chronic infection itself may be associated with worse outcomes, the observed absence of a statistically significant difference between groups should be interpreted as observational and hypothesis-generating rather than confirmatory evidence of comparable treatment pathways.

Second, the adjustment for potential confounding factors was limited. Although several comorbidities were recorded, detailed information regarding immunosuppressive medication use, corticosteroid exposure, smoking status, nutritional status, frailty, detailed glycemic control parameters, medication adherence, and other systemic host factors was not systematically available. These unmeasured variables may have influenced infection eradication and treatment outcomes. In addition, post-discharge variables such as rehabilitation participation, social support, living environment, ambulatory status, and functional recovery were not systematically assessed, despite their potential influence on long-term clinical outcomes and reinfection risk.

Third, the relatively small sample size, particularly in the F-DAIR group (*n* = 23), and the limited number of failure events (*n* = 7 total) reduced statistical power, increased the possibility of type II error, and limited the robustness of multivariable modeling. The Cox regression model may have been susceptible to overfitting and unstable hazard ratio estimates, particularly for variables with low event frequencies. Therefore, the absence of statistically significant differences between groups should not be interpreted as definitive evidence of equivalence.

Additional limitations include the inclusion of DAIR procedures performed at outside institutions, variability in follow-up methods, the possibility of missed late failures, lack of functional outcome or patient-reported outcome measures, the single-institution design, absence of detailed cost-effectiveness or resource-utilization analyses, and incomplete information regarding antibiotic protocols, including specific agents, duration, and adherence. Future prospective multicenter studies should incorporate standardized treatment protocols, more comprehensive patient-level confounder assessment, and post-discharge functional recovery data. In addition, DAIR procedures in the present study were heterogeneous and included arthroscopic debridement, open debridement, and open debridement with polyethylene liner exchange. These differences may influence bacterial burden reduction and biofilm eradication. Compared with arthroscopic procedures, open DAIR with modular component exchange may allow more thorough synovectomy, improved access to posterior compartments, and more effective reduction of biofilm burden. Furthermore, delayed conversion from failed DAIR to component removal may theoretically allow further biofilm maturation, soft-tissue compromise, and emergence of resistant organisms. Because of the relatively small sample size and limited number of failure events, subgroup analyses according to DAIR timing or surgical technique were not adequately powered and therefore were not performed.

## 5. Conclusions

This retrospective cohort study found that prior failed DAIR was not associated with significantly worse outcomes after subsequent two-stage revision arthroplasty for PJI following TKA. However, given the relatively small sample size, limited number of failure events, baseline differences between groups, and retrospective design, these findings should be interpreted cautiously and should not be considered definitive evidence of equivalence. Larger multicenter studies with standardized DAIR protocols, detailed timing data, and functional outcomes are needed to further clarify the relationship between failed DAIR and subsequent revision outcomes. Future studies should also include more comprehensive patient-level confounder assessment, standardized adjustment strategies, and post-discharge functional recovery data. In particular, the imbalance in infection chronicity and the limited ability to adjust for unmeasured host and post-discharge factors preclude causal conclusions from this cohort.

## Figures and Tables

**Table 1:** Definition of treatment outcomes according to the MSIS outcome reporting tool.

Tier	Definition
Tier 1.	Infection control with no continued antibiotic therapy
Tier 2.	Infection control with the patient on suppressive antibiotic therapy
Tier 3.	Need for reoperation and/or revision and/or spacer retention (assigned to subgroups A, B, C, D, E, and F based on the type of reoperation) A. Aseptic revision at >1 year from initiation of PJI treatment B. Septic revision (including debridement, antibiotics, and implant retention [DAIR]) at >1 year from initiation of PJI treatment (excluding amputation, resection arthroplasty, and arthrodesis) C. Aseptic revision at ≤1 year from initiation of PJI treatment D. Septic revision (including DAIR) at ≤1 year from initiation of PJI treatment (excluding amputation, resection arthroplasty, and arthrodesis) E. Amputation, resection arthroplasty, or arthrodesis F. Retained spacer
Tier 4.	Death (assigned to subgroups A or B). A. Death ≤ 1 year from initiation of PJI treatment B. Death > 1 year from initiation of PJI treatment

**Table 2:** Demographic and clinical characteristics of the study groups.

	F-DAIR Group (*n* = 23)	Failure (*n* = 1)	DTSR Group (*n* = 61)	Failure (*n* = 6)	*p* Value	Overall (*n* = 84)
Age (Mean, range) (years)	69.78 (38–84)		71.64 (48–83)		0.409	71.13 (±8.16)
>70 years	13 (56.5%)		17 (27.9%)	3	0.172	
Gender					0.271	
Male	8 (34.8%)		14 (23.0%)	3		22 (26.2%)
Female	15 (65.2%)	1	47 (77.0%)	3		62 (73.8%)
ASA grade					0.3	
ASA 1	1 (4.3%)		7 (11.5%)	2		8 (9.5%)
ASA 2	16 (69.6%)		46 (75.1%)	3		62 (73.8%)
ASA 3	6 (26.1%)	1	8 (13.1%)	1		14 (16.7%)
ASA 4	0		0			0
Body mass index (BMI)	24.39 (17.3–31.1)		25.50 (17.58–41.26)		0.228	24.69 (±3.75)
Co-morbidities						
Diabetes	11 (47.8%)	1	20 (32.8%)	3	0.203	31 (36.9%)
Rheumatoid Arthritis	1 (4.3%)		3 (4.9%)	1	0.913	4 (4.8%)
COPD	0		4 (6.6%)	0		4 (4.8%)
Chronic Kidney Disease	0		2 (3.3%)	0		2 (2.4%)
Liver Cirrhosis	2 (8.7%)	1	2 (3.3%)	0	0.301	4 (4.8%)
Cardiovascular Disease	4 (17.4%)		11 (18.0%)	1	0.945	15 (17.9%)
Infection Type					0.001 *	
Acute postoperative	10 (43.5%)		7 (11.5%)	2		17 (20.2%)
Acute hematogenous	9 (39.1%)	1	22 (36.1%)	3		31 (36.9%)
Chronic	4 (17.4%)		32 (52.5%)	1		36 (42.9%)
Implant age (month)	26.65 (1–173)		45.07 (2–203)		0.097	40.54 (±45.067)
Stage interval (days)	65.23 (27–279)		56.34 (40–101)		0.149	62.80 (±34.39)
Follow-up periods (Month)	57.17 (16–136)		63.51 (1–178)		0.457	61.77 (±40.485)

The asterisk (*) after the value was used to indicate statistical significance at the 0.05 level.

**Table 3:** Microbiological profiles of the study groups.

	F-DAIR Group (*n* = 23)	Failure (*n* = 1)	DTSR Group (*n* = 61)	Failure (*n* = 6)	*p* Value	Overall (*n* = 84)
Culture negative	4 (17.4%)		33 (54.1%)	3	0.003 *	37 (44.0%)
Antibiotic-resistant bacteria	11 (47.8%)		8 (13.1%)	1	0.001 *	
MRSE (*Staphylococcus epidermidis*)	9 (39.1%)		4 (6.6%)		0.001 *	13 (15.5%)
MRSA (*Staphylococcus aureus*)	2 (8.7%)		4 (6.6%)	1	0.663	6 (7.1%)
*Escherichia coli*	2 (8.7%)	1	4 (6.6%)		0.663	6 (7.1%)
MSSA (*Staphylococcus aureus*)	1 (4.3%)		4 (6.6%)		1.00	5 (6.0%)
*Streptococcus dysgalactiae*	1 (4.3%)		2 (3.3%)	1	1.00	3 (3.6%)
*Streptococcus pneumoniae*	1 (4.3%)		0		0.274	1 (1.2%)
*Candida* spp.	1 (4.3%)		2 (3.3%)		1.00	3 (3.6%)
*Acinetobacter baumannii*	1 (4.3%)		0		0.274	1 (1.2%)
*Mycobacterium tuberculosis*	0		2 (3.3%)		1.00	2 (2.4%)
*Klebsiella pneumoniae*	0		1 (1.6%)		1.00	1 (1.2%)
*Pseudomonas* spp.	0		1 (1.6%)		1.00	1 (1.2%)
Coagulase-negative staphylococci	0		1 (1.6%)	1	1.00	1 (1.2%)
*Enterobacter cloacae*	1 (4.3%)		0		0.274	1 (1.2%)
*Enterococcus faecalis*	0		1 (1.6%)		1.00	1 (1.2%)
*Corynebacterium striatum*	0		1 (1.6%)		1.00	1 (1.2%)
MSSE (*Staphylococcus epidermidis*)	0		1 (1.6%)		1.00	1 (1.2%)

* Statistically significant (*p* < 0.05).

**Table 4:** Treatment outcomes according to the MSIS classification.

	F-DAIR Group (*n* = 23)	DTSR Group (*n* = 61)	Overall (*n* = 84)
Tier 1	20 (87.0%)	46 (75.4%)	66 (78.6%)
Tier 3B	0	2 (3.3%)	2 (2.4%)
Tier 3D	1 (4.3%)	2 (3.3%)	3 (3.6%)
Tier 4A	0	2 (3.3%)	2 (2.4%)
Tier 4B	2 (8.7%)	9 (14.8%)	11 (13.1%)

**Table 5:** Cox proportional hazards regression analysis for predictors of treatment failure.

Covariate	Comparison	HR	95% CI	*p*-Value
DAIR	F-DAIR vs. DTSR	0.043	0.002–1.039	0.053
Age	Age < 70 vs. Age > 70	0.751	0.077–7.286	0.805
PJI type	Acute vs. chronic	0.028	0.002–0.527	0.017 *
Culture	negative vs. positive Non-resistant vs. Antibiotic-resistant	0.939	0.055–15.889	0.965
Diabetes	No diabetes vs. diabetes	12.809	1.217–134.769	0.034
Rheumatoid Arthritis	No RA vs. RA	3.392	0.249–46.171	0.359
Liver Cirrhosis	No LC vs. LC	33.725	1.325–858.530	0.098
Age of prosthesis		0.986	0.938–1.037	0.590
ASA score		2.412	0.249–46.171	0.410
BMI		1.094	0.818–1.463	0.545

The asterisk (*) after the value was used to indicate statistical significance at the 0.05 level.

## Data Availability

The data that support the findings of this study are not publicly available due to restrictions related to patient privacy and institutional regulations. De-identified data may be available from the corresponding author upon reasonable request and with approval from the Institutional Review Board (IRB).
